# Plasma LOX-Products and Monocyte Signaling Is Reduced by Adjunctive Cyclooxygenase-2 Inhibitor in a Phase I Clinical Trial of Tuberculosis Patients

**DOI:** 10.3389/fcimb.2021.669623

**Published:** 2021-07-09

**Authors:** Marthe Jøntvedt Jørgensen, Kristin G. Nore, Hans Christian D. Aass, Emilie Layre, Jérôme Nigou, Rasmus Mortensen, Kjetil Tasken, Dag Kvale, Synne Jenum, Kristian Tonby, Anne Ma Dyrhol-Riise

**Affiliations:** ^1^ Institute of Clinical Medicine, University of Oslo, Oslo, Norway; ^2^ Department of Infectious Diseases, Oslo University Hospital, Oslo, Norway; ^3^ Department of Medical Biochemistry, Oslo University Hospital, Oslo, Norway; ^4^ Institut de Pharmacologie et de Biologie Structurale, Université de Toulouse, CNRS, Université Paul Sabatier, Toulouse, France; ^5^ Department of Infectious Disease Immunology, Statens Serum Institut, Copenhagen, Denmark; ^6^ Deparment of Cancer Immunology, Institute for Cancer Research, Oslo University Hospital, Oslo, Norway

**Keywords:** host-directed therapy (HDT), eicosanoids, cyclooxygenase-2 inhibitor, tuberculosis, monocytes, cytokines, innate immunity, lipooxygenase

## Abstract

**Introduction:**

Eicosanoids and intracellular signaling pathways are potential targets for host-directed therapy (HDT) in tuberculosis (TB). We have explored the effect of cyclooxygenase 2 inhibitor (COX-2i) treatment on eicosanoid levels and signaling pathways in monocytes.

**Methods:**

Peripheral blood mononuclear cells isolated from TB patients included in a randomized phase I clinical trial of standard TB treatment with (n=21) or without (n=18) adjunctive COX-2i (etoricoxib) were analyzed at baseline, day 14 and day 56. Plasma eicosanoids were analyzed by ELISA and liquid chromatography-mass spectrometry (LC-MS), plasma cytokines by multiplex, and monocyte signaling by phospho-flow with a defined set of phospho-specific antibodies.

**Results:**

Lipoxygenase (LOX)-derived products (LXA4 and 12-HETE) and pro-inflammatory cytokines were associated with TB disease severity and were reduced during TB therapy, possibly accelerated by adjunctive COX-2i. Phosphorylation of p38 MAPK, NFkB, Erk1/2, and Akt in monocytes as well as plasma levels of MIG/CXCL9 and procalcitonin were reduced in the COX-2i group compared to controls.

**Conclusion:**

COX-2i may reduce excess inflammation in TB *via* the LOX-pathway in addition to modulation of phosphorylation patterns in monocytes. Immunomodulatory effects of adjunctive COX-2i in TB should be further investigated before recommended for use as a HDT strategy.

## Introduction

Tuberculosis (TB), caused by *Mycobacterium tuberculosis* (*Mtb)* is responsible for an estimated 1.5 million deaths annually (WHO, 2020). Although a curable disease, effective TB treatment is challenged by increasing incidence of multi-drug resistant TB (MDR-TB). Host-directed therapy (HDT) has emerged as an alternative treatment strategy, aiming to increase treatment efficacy and shorten treatment duration by modulation of host immunity (Kolloli and Subbian, 2017).

**Figure 1:**
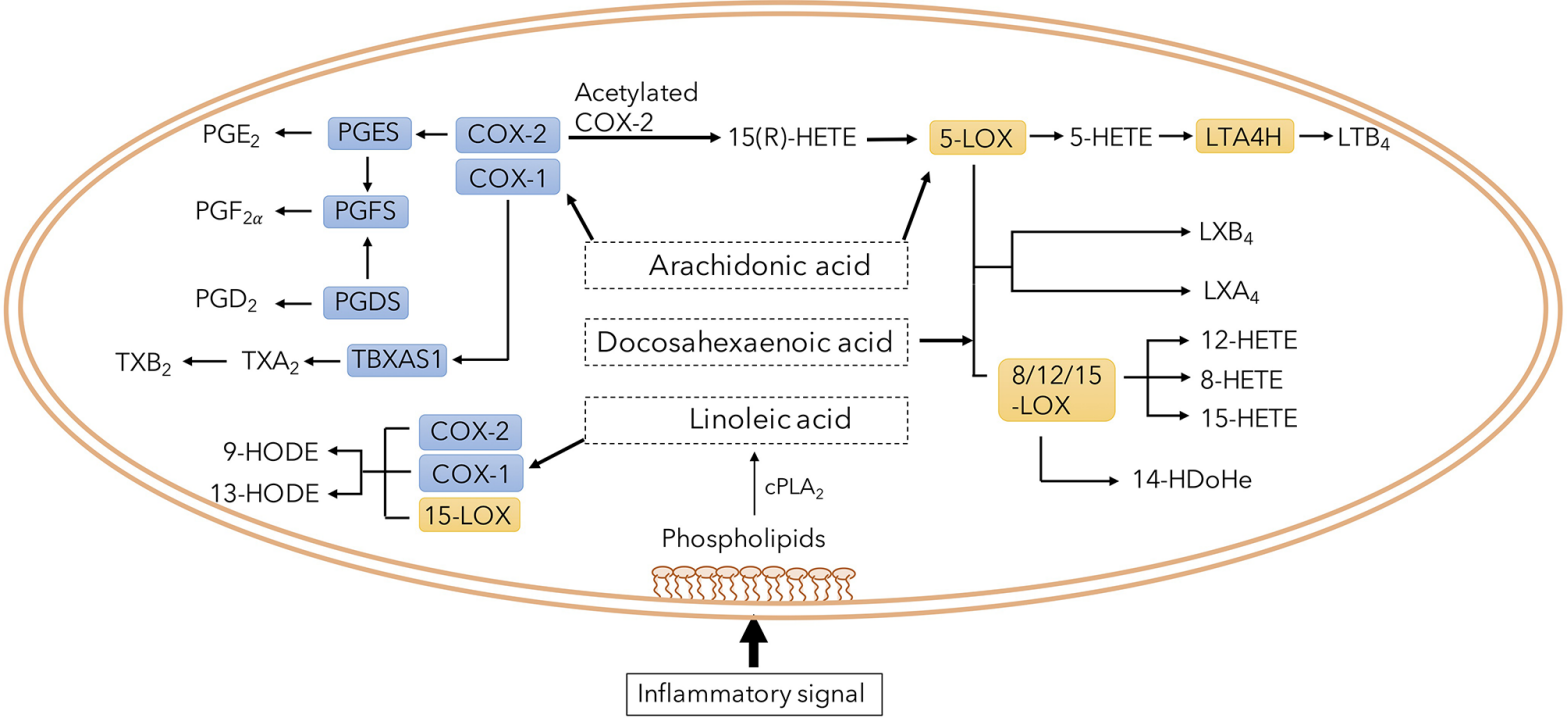
Overview of eicosanoid pathway and its metabolites. Phospholipids in the plasma membrane is converted to several specific fatty acids. Among them, arachidonic acid is a common substrate for both COX and LOX enzymes generating immune modulatory prostaglandins, lipoxins and leukotrienes.

The eicosanoid system, encompassing several biologically active lipid mediators, have been proposed to play an important role in the pathophysiology of *Mtb* infection (Peres et al., 2007; Dennis and Norris, 2015; Sorgi et al., 2020). Their synthesis is predominantly regulated by two families of intracellular enzymes, Cyclooxygenase (COX) and Lipoxygenase (LOX), of which there are several different subclasses. COX-2 is upregulated by inflammation and generates prostanoids including prostaglandin E2 (PGE2) while 5-LOX, 12-LOX, 15-LOX and 8-LOX produce lipoxins, leukotrienes and intermediate metabolites such as hydroxyeicosatetraenoic acids (HETEs) (**Figure 1**) (Dennis and Norris, 2015). Recent reports highlight a dysregulation of the eicosanoid network, with a skewed balance toward LOX products, promoting tissue damage and mycobacterial survival (Chen et al., 2008; O’connor et al., 2016; Pedruzzi et al., 2016). Lipoxin A4 (LXA4) seems to induce macrophage death while 12/15-HETE drive neutrophilic inflammation with subsequent tissue damage (Chen et al., 2008; Mishra et al., 2017), but effects of other products of the LOX pathway in TB pathogenesis are unclear.

Approved drugs that augment PGE2 levels have been suggested as a possible HDT-strategy in TB as PGE2 has been reported to limit detrimental type I interferon (IFN)-production in *Mtb* infected mice (Mayer-Barber et al., 2014) and induce macrophage apoptosis rather than necrosis (Divangahi et al., 2013). However, excess levels of PGE2 may also contribute to disease progression by inhibiting cell-mediated immunity (Rangel Moreno et al., 2002). COX-2 inhibitors (COX-2i) that inhibit PGE2 production reduce bacterial burden and increase survival in some animal models (Vilaplana et al., 2013; Sorgi et al., 2020), although the effect of COX-2i seems to be dependent on route of infection (Mortensen et al., 2019). Thus, COX-2i is of interest as potential HDT.

Monocytes and macrophages are key cellular players in TB pathogenesis and major producers of inflammatory mediators and eicosanoids (Rangel Moreno et al., 2002; Chen et al., 2008; Dennis and Norris, 2015). Toll like receptors (TLRs) and other bacterial pattern recognition receptors on the cell surface recognize foreign pathogens and initiate downstream signaling, resulting in the initiation of the early immune responses. Upon binding to the receptor, protein phosphorylation induces a set of transcription factors leading to production of pro-inflammatory cytokines such as PGE2, TNF- α, IL-12, IL-1 and IL-6 (Guha and Mackman, 2001; Basu et al., 2012).

The signaling pathways of p38 mitogen-activating protein kinase (MAPK), inhibitory κB kinase (IkK) and nuclear factor-κB (NFκB), interferon regulatory factors (IRF) and extracellular signal-regulated kinase (ERK) seem to be involved in monocyte/macrophage-derived cytokine signaling in response to mycobacterial antigens (Barnes and Karin, 1997; Zingarelli et al., 2003; Jo et al., 2007). *Mtb* infected macrophages produce high levels of PGE_2_, partly mediated through TLR2/p38 MAPK signaling, thereby inducing apoptosis of *Mtb* infected macrophages (Nishimura et al., 2013). Translocation of NFκB to the nucleus and transcription of pro-inflammatory genes plays a key role in TB control, and NFκB has been suggested as a possible therapeutic target (Zingarelli et al., 2003; Tay et al., 2010; Fallahi-Sichani et al., 2012; Bai et al., 2013). During TB infection, eicosanoids seem to exert immunomodulatory functions by affecting the production of cytokines such as IL-1, IFN-γ and TNF-α (Tobin et al., 2010; Braverman et al., 2016; Cadena et al., 2016) through altered intracellular signaling, hereby representing a potential HDT target (Almeida et al., 2014). However, the effects of COX-2i treatment on eicosanoid production and monocyte signaling during TB disease remain unknown.

To obtain more insight into eicosanoid biology in TB we first studied the association between different eicosanoid mediators and severity of TB disease. To further evaluate the potential of COX-2i as HDT we investigated the effects of COX-2i on eicosanoid and cytokine levels in plasma from TB patient recruited into a phase I/II clinical trial assessing the safety and immunogenicity of adjunctive COX-2i in TB disease (TBCOX2 study). Finally, to explore novel HDT targets related to innate immunity we analyzed various signaling pathways in monocytes and the *in vitro* and *ex vivo* effects of COX-2i on signal transduction.

## Materials and Methods

### Study Participants

Samples were collected from a total of 39 patients with culture confirmed drug sensitive TB recruited from a phase I/II/clinical trial at Oslo University Hospital, Norway in the period 2015-2019 (TBCOX2, *NCT02503839*). 18 patients received adjunctive COX-2i treatment (etoricoxib) for 140 days in addition to standard TB treatment and 21 patients received only standard TB treatment (**Table 1**). All participants experienced clinical improvement and culture conversion after 2 months of treatment. In addition, five patients (age 18-70) with pulmonary TB were included in a pilot study with blood sampling before TB treatment initiation. All participants were HIV negative. Clinical examination, symptoms, analyses of erythrocyte sedimentation rates (ESR), monocytes and lymphocytes (ML) ratio in peripheral blood, and chest X-ray performed at baseline were recorded. For an overview of the patients included in the different assays see **Supplementary Figure S1**.

**Table 1:** Patient characteristics.

	Total (n = 39)	COX-2i (n = 18)	Control (n = 21)
Age (median)	27 (18-52)	29 (19-49)	26 (18-52)
Male (%)	21 (54)	9 (50)	12 (57)
**Origin**			
Black	20 (51)	8 (44)	12 (57)
Asian	11 (28)	5 (28)	6 (29)
Caucasian	6 (15)	3 (17)	3 (14)
Other	2 (5)	2 (11)	0
**Clinical presentation**			
Pulmonary	28^a^	14^b^	16^c^
Cavity	9	5	4
Extrapulmonary	7	4	3
**Symptoms**			
Cough (%)	20 (51)	10 (56)	10 (48)
Night-Sweat (%)	18 (46)	7 (39)	11 (52)
Weight loss (%)	15 (39)	9 (50)	6 (29)
Fever (%)	9 (23)	5 (28)	4 (19)
Chest pain (%)	11 (28)	4 (22)	7 (33)
Low:high symptom score^d^	17:22	8:10	9:12
**Findings**			
BMI^e^ (min-max)	21 (16-30)	21 (16-30)	21 (17-27)
ML ratio^f^ (min-max)	0.33 (0.13-1.36)	0.36 (0.13-1.36)	0.33 (0.17-1.14)
ESR^g^ (mm/hour, min-max)	20 (1-116)	26 (2-105)	20 (1-116)
TTP^h^ (min-max)	12.2 (2.7-42.1)	12.8 (2.71-24.9)	12.2 (4.7-42.1)
Ct values^i^ (min-max)	36 (31-46)	34 (31-46)	41 (31-46)

a 4/28, ^b^2/14 and ^c^2/16 with both PTB and EPTB.

d High =≥2 of the following symptoms: Cough, night-sweat, weightloss and fever (>38°C). Low = 1 symptom or asymptomatic/detected by screening.

e Body mass index (n= 34).

f Myeloid:lymphocyte ratio (n = 34).

g Erythrocyte Sedimentation Rate (n=34).

h Time to Mtb positive culture, days (n=30).

I Cycle threshold values.

### Sample Collection and Preparation

Peripheral blood was drawn at baseline, day 14 and day 56. Blood samples were collected in CPT™ Cell Preparation tube (BD Biosciences), using Sodium-Heparin as anti-coagulant, and immediately centrifuged 15 minutes at 1700 g. Plasma was snap-frozen and stored at -80°C until analysis. Peripheral blood mononuclear cells (PBMC) were isolated and frozen containing freezing media with 10% DMSO. Sputum or relevant tissue specimens were incubated at 37°C for minimum 42 days in Mycobacteria Growth Indicator Tube (MGIT, BD biosciences, New Jersey, USA) and the number of days to detection of bacteria can be measured as time to positive sample (TTP). Cycle threshold (Ct) values were obtained from analysis with quantitative PCR assay (Xpert MTB/RIF) for rapid detection of *Mtb*-specific nucleic acids.

### Chemicals and Reagents

Commercially available EIA kits were used to measure PGE2 (cat.no. 514010, Cayman chemical, Ann Harbour, Michigan, USA) and LXA4 (cat.no. EA45 Oxford Biomedical Research, Oxford, Michigan, USA). Samples underwent extraction protocols using C18-SPE Cartridges (Cat.no WAT023501, Waters Inc, Massachusetts, USA) prior to EIA analysis. Cytokines were analyzed using Magnetic Luminex assay (cat.no. LXSAHM-24, RnD systems, Minneapolis, Canada and SAA Human ProCartaPlex™ Simplex Kit (cat.no. EPX01A-12136-901, Thermo Fisher Scientific, Massachusetts, USA)

Directly conjugated monoclonal antibodies for staining monocyte surface markers were directed to HLA-DR FITC (cat.no. 307604, Biolegend, San Diego, USA) and anti-CD14 PE antibodies (cat.no. 345785, BD Bioscience), antibodies for intracellular phosphoflow staining were anti – p38 mitogen activated protein kinase (MAPK) (pS180/S182) (cat.no. 612595), extracellular signal-regulated kinase (ERK) 1/2 (pT202/pY204) (cat.no. 6125939), Protein kinase B (Akt) (pS473) (cat.no. 560343), Nuclear factor κB (NFκB) p65 (pS529) (cat.no. 5584229, interferon regulatory factor (IRF)-7 (pS477) (cat.no. 558630), Cyclic AMP- response element binding protein (CREB) (pS133) ATF-1 (pS63) (cat.no. 558434), protein kinase A (PKA) RIIb (pS114) (cat.no. 560205) all from BD, Biosciences, San Jose, CA, USA. Fluorescent cell barcoding reagents were Pacific Blue Succinimidyl Ester (cat.no. P10163, Thermo Fisher Scientific, Massachusetts, USA) and Pacific Orange Succinimidyl Ester (cat.no. P30253, Thermo Fisher Scientific). Cells were fixed and permeabilized using BD Phosphoflow™ Fix Buffer I (BD Bioscience, cat.no. 557870) and BD Perm/Wash (BD Bioscience, cat.no. 554723).

COX-1/2 inhibitor used in the *in vitro* signaling assay were Indomethacin (20uM, cat.no. I7378-100G, Sigma Aldrich, Saint Louis, Missouri, USA). Cells were counted using Trypan Blue Solution 0.4% (cat.no. 15250061, Gibco™, Thermo Fisher Scientific) and stimulated with either 10ug/mL PPD (SSI, Denmark), 10ng/mL lipopolysaccharide (LPS) or 10mM Prostaglandin E2 (cat.no. HY101952, MedChemExpress, New Jersey, USA).

### Enzyme-Linked Immunosorbent Assay

Using a competitive parameter immunoassay, human plasma concentrations of PGE2 and LXA4 from TB patients treated with or without adjunctive COX-2i, at diagnosis and day 14 was quantified using commercial EIA. All assays were performed according to manufacturer’s instructions. Briefly, samples underwent extraction protocols using C18-SPE Cartridges. Samples were run in duplicates and optical density was determined at 450 nm or 650 nm using a Spectramax Abs plus microplate reader (Molecular devices Corporation).

### Liquid Chromatography – Mass Spectrometry

Eicosanoid concentrations in plasma from patients treated with or without adjunctive COX-2i were analyzed at baseline, day 14 and day 56. Quantification of 5 – hydroxyeicosatetraenoic acid (HETE), 8-, 12 and 15 – HETE, 9-hydroxyoctadecadienoic acid (HODE), 13 – HODE, 14 Hydroxydocosahexaenoic acid (HDoHe) using liquid chromatography – mass spectrometry (LC-MS) was performed as previously described (Le Faouder et al., 2013) at the MetaToul Lipidomic Core Facility (I2MC, Inserm 1048, Toulouse, France, MetaboHUBANR-11-INSB-0010). In the panel, 19 metabolites were not detectable in plasma [resolvin (RV) E1, D1, D2, D3, D5, thromboxane B2, 11B-prostaglandin (PG) F2a, PGE3, PGF2a, PGE2, PGD2, PGA1, 8-iso-PGA2, 6-keto PGF1a, 15-deoxy-delta PGJ2, LXB4, LXA4, LTB5, 7-Maresin 1, 18-hyrdoxyeicosapentanoic acid (HEPE), 5,6 DiHETE, 17-HDoHe, 14,15-epoxy eicosatrienoic acid (EET), 5-oxo-EET, 11,12-EET, 8,9-EET, 5,6-EET]. Briefly, methanol and internal standard (Deuterium labeled compounds) was added before centrifugation (2000 g for 15 min at 4°C). Supernatants were transferred into 96-well deep plates and diluted in H2O. Samples were then submitted to solid phase extraction (SPE) using OASIS HLB 96-well plate (30 mg/well, Waters) and reconstituted in MeOH. Lipid mediators were separated on a ZorBAX SB-C18 column (Agilent Technologies) using Agilent 1290 Infinity HPLC system (Technologies) coupled to an ESI-triple quadruple G6460 mass spectrometer (Agilent Technologies). Data were acquired in Multiple Reaction Monitoring (MRM) mode with optimized conditions (ion optics and collision energy). Peak detection, integration and quantitative analysis were done using Mass Hunter Quantitative analysis software (Agilent Technologies) based on calibration lines built with commercially available eicosanoids standards (Cayman Chemicals). Metabolites that were not detectable in more than 30% of the samples were excluded for further analysis.

### Cytokine Analysis

Measurements of cytokines in plasma collected from the COX-2i and control groups at baseline, day 14 and day 56 were performed using a Magnetic Luminex assay with a Luminex IS200 instrument (Bio-Rad). Measurements of chemokine (C-C motif) ligand 1 (CCL1), macrophage inflammatory protein-1*α* (MIP-1*α*/CCL3), MIP-1*β*/CCL4, interferon (IFN) IFN-*α*, IFN-*β*, IFN-*γ*, macrophage-derived chemokine (MDC/CCL22), Monokine induced by gamma (MIG/CXCL9), granulocyte colony stimulating factor (G-CSF), monocyte chemoattractant protein-1 (MCP-1/CCL2), interleukin (IL)-1*β* /IL-1F2, IL-2, IL-12p70, IL-1r*α*, IL-4r*α*, IL-8/CXCL8, IL-18/IL-1F4, CD25/IL-2r*α*, pentraxin 3, S100 calcium-binding protein A9 (S100A9), IFN-*γ* inducible protein (IP-10/CXCL10), matrix metalloproteinase-1 (MMP-1), procalcitonin and tumor necrosis factor (TNF)-*α* were analyzed using 24-plex kit while serum amyloid A(SAA) were analyzed using ProCartaPlex. Analyses were performed in duplicates and analyzed with the Bio-Plex manager Software version 6.2 (build 175). Out of range values (OOR)> were set to the highest measurable concentration and OOR< were set to zero. Values that were out of the standard range but stipulated from the standard curve were included. Levels of IFN-*α*, IFN-*β*, IFN-*γ*, MIP-1*β*/CCL4, IL-12p70 were not detectable in more than 30% of the samples and were therefore excluded for further analysis

### Cell Culture

Cryopreserved PBMCs were thawed and rested for 1h in a 37°C w/5% CO_2_ incubator. Thawed cells were manually counted by microscope, and viability was measured by using Trypan Blue Solution. All samples included had a viability above 80% and the majority of the samples had a viability above 90%. Next, cells were subjected to various stimulation conditions ranging from 0 to 60 min. In a pilot study, PBMCs from confirmed pulmonary TB patients were collected before initiation of TB treatment. Cells were either unstimulated or stimulated with 10ng/mL LPS, 10ug/mL PPD or PPD in combination with 20uM Indomethacin and immediately fixed at 0 min, 10, 30 and 60 min after stimulation. Indomethacin was added to cells 30 min prior to PPD stimulation. PBMCs from the TBCOX2 clinical trial (COX-2i group, n= 8, controls, n=6) were subjected to the same procedures as the pilot study and stimulated with either 10ng/mL LPS, 10ug/mL PPD or 10uM PGE2 for the same period of time, but with no addition of Indomethacin to cultures.

### Flow Cytometry

Phosphoflow were performed as previously described (Hermansen et al., 2018; Skanland, 2018). Briefly, prior to permeabilization, the different stimuli conditions were barcoded with different combinations of Pacific Blue and Pacific Orange in room temperature for 20 min. After barcoding, cells were washed with PBS containing 2%FBS, pooled, permeabilized and stained with anti-HLA-DR FITC and anti-CD14 PE antibodies (BD Bioscience) and 6 different phospho – specific antibodies namely anti – p38 mitogen activated protein kinase (MAPK) (pS180/S182), extracellular signal-regulated kinase (ERK) 1/2 (pT202/pY204), Protein kinase B (Akt) (pS473), Nuclear factor κB (NFκB) p65 (pS529), interferon regulatory factor (IRF)-7 (pS477), Cyclic AMP- response element binding protein (CREB) (pS133) ATF-1 (pS63) and in samples from TB patients treated with or without adjunctive COX-2i: Protein kinase A (PKA) RIIb (pS114) was included. **Supplementary Table S1** display antibodies included in the experiments. After 30 min incubation, cells were subjected to flow cytometry analysis with BD FACS Canto II. Monocytes were defined as HLA-DR^+^ and CD14^+^, the gating strategy can be found in **Supplementary Figure S2**. Cell acquisition (<300,000 events) was performed on a FACS Canto II (BD Biosciences). Instrument calibration was performed according to manufacturer’s instructions and compensation settings adjusted using antibody-capture beads (CompBeads, BD Biosciences).

### Statistical Analysis

For plasma analytes, all data are expressed as median and interquartile range (IQR). Non-parametrical statistical methods were applied, Mann-Whitney U test was used for unpaired data and Wilcoxon for matched pairs, Spearman for correlation analysis. Due to the exploratory nature of the data, it was not corrected for multiple comparisons, but cation was taken when interpreting the results. For flow cytometry analysis, the pooled stimulated samples could be deconvoluted with the different barcoding signatures and analyzed individually. Samples were analyzed using Cytobank (https://cellmass.cytobank.org) and Graphpad Prism (LCC, San Diego, US) and phosphorylation intensities are displayed as arcsinh ratio of medians. Mann U Whitney test was used to compare two unrelated groups. Multiple comparison with Holm Sidak’s correction was used to compare phosphorylation time courses between the control and COX-2i group.

## Results

### Lipoxygenase (LOX)-Derived Metabolites Are Elevated in Cavitary TB Disease

To investigate the role of eicosanoid metabolites in TB pathology, we stratified our cohort based on clinical criteria for disease severity at diagnosis (**Table 1**). In our cohort of 39 TB patients, 23% (n=9) displayed pulmonary TB with cavitary disease and 18% (n=7) were defined as extrapulmonary TB. A total of 22 patients had a high symptom score while 17 patients had low symptom score. Clinical parameters such as erythrocyte sedimentation rate (ESR), monocyte-lymphocyte (ML) ratio, time-to-positive *Mtb* culture (TTP) and cycle threshold (Ct) values were comparable in both groups at diagnosis.

**Figure 2:**
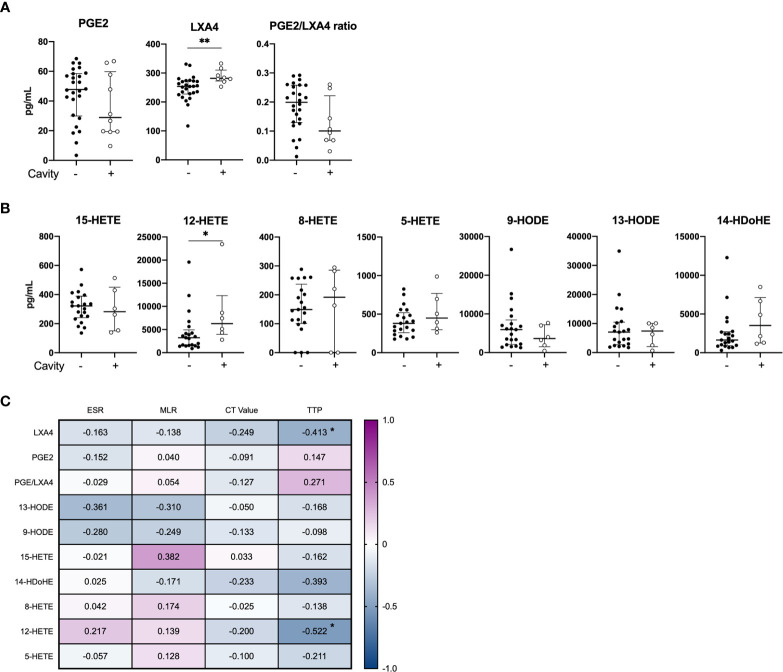
Baseline plasma eicosanoid profiles in cavitary vs. non-cavitary TB disease and correlations to clinical markers. Plasma levels of **(A)** PGE2, LXA4 and PGE2/LXA4 ratio displaying cavitary (n = 8) and non-cavitary disease (n = 26) in TB patients at diagnosis measured by ELISA (included 16 patients in the control group and 18 patients in the COX-2i group) and **(B)** Eicosanoid metabolites comparing cavitary (n=6) and non-cavitary disease (n = 21) in TB patients at diagnosis measured by LC-MS (included 10 patients in control group and 18 patients in COX-2i group). One baseline sample was excluded due to limited plasma. **(C)** Eicosanoid correlations to clinical parameters erythrocyte sedimentation rate (ESR), monocyte lymphocyte (ML) ratio, Cycle threshold (CT) values and time to positive *Mtb* culture (TTP) collected at diagnosis. Significance calculated with Mann Whitney T test, *p < 0.05, **p < 0.01, Lines indicate median with interquartile range (IQR). Rho calculated with spearman correlation.

While there was no difference in the levels of PGE2 between the clinical groups at baseline, mediators of the LOX pathway were elevated (LXA4, p=0.006 and 12-HETE, p=0.042) in cavitary disease compared to non-cavitary disease (**Figure 2**). No difference in eicosanoid concentrations were found when patients were stratified by symptom score (**Supplementary Figure S3**). PGE2 levels did not correlate with any laboratory markers of disease severity (ESR, ML ratio, TTP and Ct values), whereas LXA4 (r = -0.413, p=0.052) and 12-HETE (r = -0.522, p = 0.018) correlated inversely with time to positive *Mtb* culture (TTP) (**Figure 2C**).

### Lipoxygenase (LOX)-Derived Products Decline With Adjunctive COX-2i Treatment

**Figure 3:**
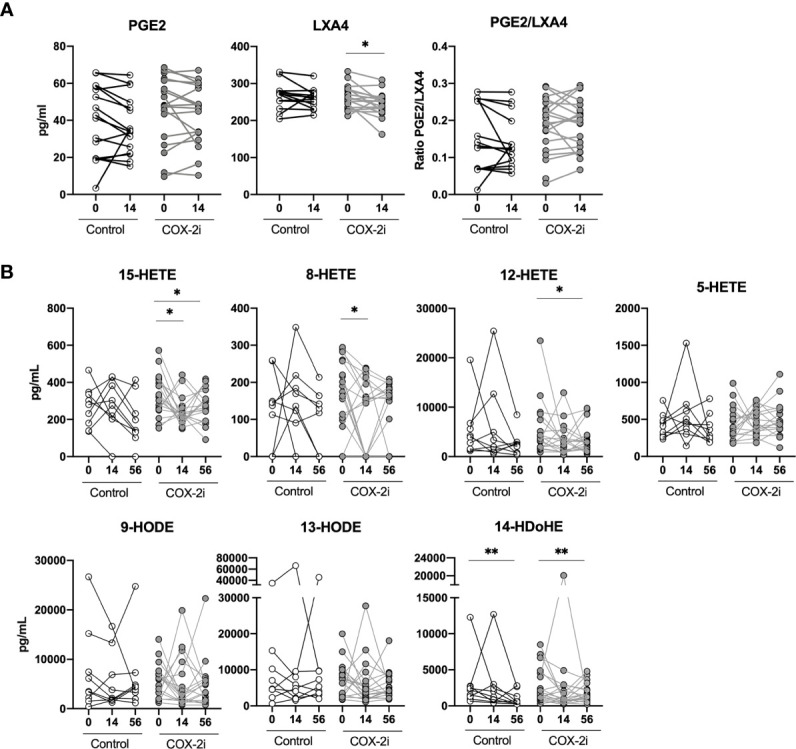
Plasma eicosanoids levels during standard TB therapy alone and with adjunctive COX-2i. **(A)** Plasma levels of PGE2, LXA4 and PGE2/LXA4 ratio measured by ELISA comparing 14 days of treatment without (n = 16) and with (n = 18) COX-2i therapy. **(B)** eicosanoid metabolites measured by LC-MS at diagnosis, 14 and 56 days after treatment without (open circles, n = 10) and with (grey circles, n = 18) COX-2i. Significance calculated with Wilcoxon test comparing baseline and day 14 and baseline and day 56. *p < 0.05, **p < 0.01. Lines indicate median with interquartile range (IQR).

To explore possible effects of adjunctive COX-2i treatment we analyzed eicosanoid metabolites in plasma after 14 days of treatment when etoricoxib was expected to reach a steady state. Etoricoxib concentrations were detectable in plasma in all patients (data not shown). We observed no significant decline in PGE2 levels (COX-2 derived) in the COX-2i-group nor in controls (**Figure 3A**). By contrast, LXA4 levels (5-LOX-derived) declined significantly (p = 0.024) in the COX-2i-group but not in controls. Although the median PGE2/LXA4 ratio was higher in the COX-2i group at baseline, no significant changes were observed for any of the groups after 14 days of treatment (**Figure 3A**).

We then analyzed longitudinal effects of adjunctive COX-2i on eicosanoid plasma profiles after 14 and 56 days in more detail by LC-MS (**Figure 3B**). From diagnosis up to day 56, LOX metabolites such as 15-HETE (day 14, p = 0.034, day 56, p = 0.048), 8-HETE (day 14, p = 0.045), 12-HETE (day 56, p = 0.054) and 14-HDoHE (day 56, p = 0.01) were significantly reduced in the COX-2i-group. In controls, the only metabolite that was decreased at day 56 was 14-HDoHE (day 56, p = 0.01). Still, there were no significant differences at day 14 nor 56 when comparing the levels of metabolites between the COX-2i-group and controls at the respective time points (**Supplementary Figure S4**).

### The Adjunctive Effects of COX-2i on Plasma Cytokines During TB Treatment

**Figure 4:**
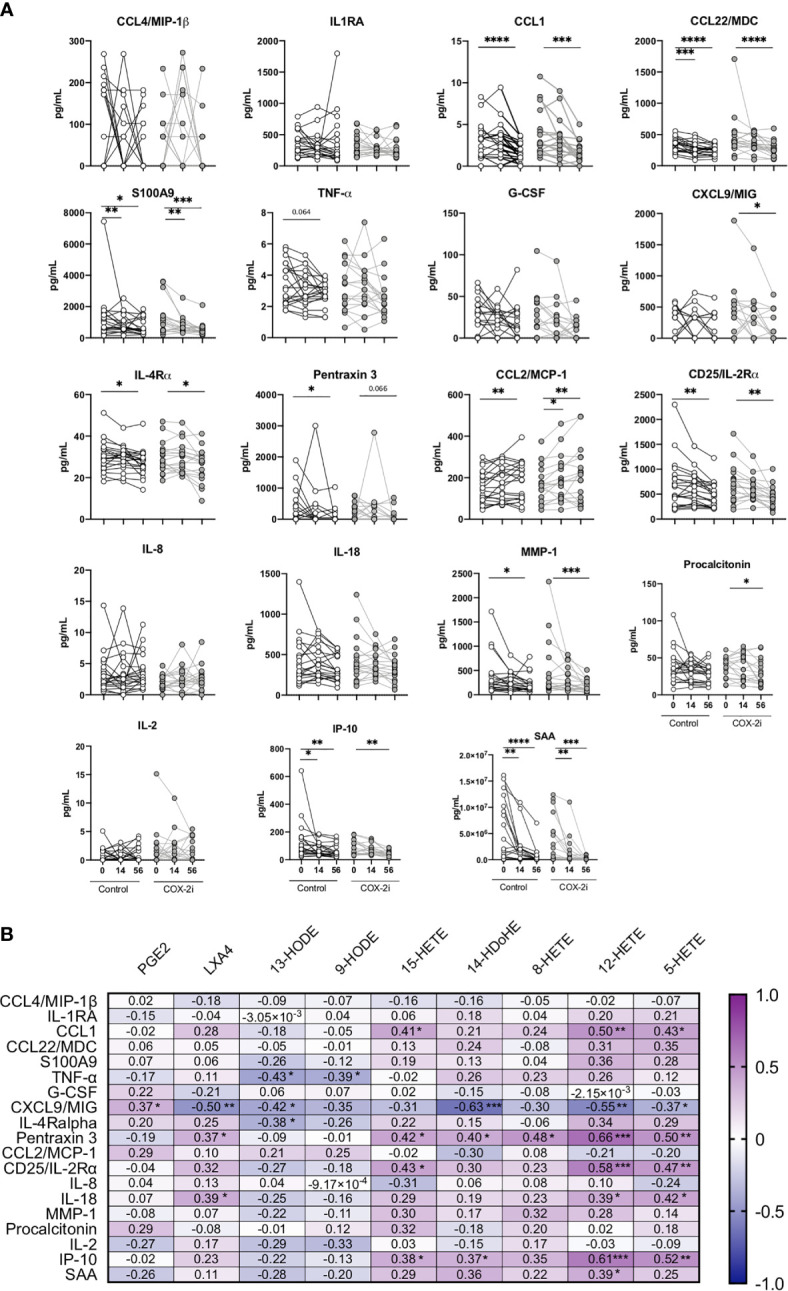
Plasma cytokine levels during standard TB therapy alone and with adjunctive COX-2i. **(A)** Levels of cytokines in plasma in TB patients without (n = 21) and with (n = 18) COX-2i therapy. Significance calculated with Wilcoxon test comparing baseline and day 14 and baseline and day 56. *p < 0.05, **p < 0.01, ***p < 0.001, ****p < 0.0001, Lines indicate median with interquartile range (IQR). **(B)** Relationship between eicosanoids and cytokines levels in plasma from TB patients at diagnosis. Correlations are displayed using the Rho-value calculated with spearman correlation.

A broad specter of plasma cytokines was screened using a 24-plex kit and a single-plex Luminex Kit. The pro-inflammatory cytokines CCL1, Pentraxin3, CD25/IL-2ra, IP-10, S100A9 and MMP-1 correlated with markers of disease severity (**Supplementary Figure S5**). As COX-2i has anti-inflammatory properties (Kroesen et al., 2017) we investigated if COX-2i treatment influenced on plasma cytokines levels. In general, the inflammatory mediators declined during TB therapy in both the COX-2i group and in controls. Still, CXCL9/MIG and procalcitonin levels were significantly reduced after 56 days only in the COX-2i-group (**Figure 4A**). In contrast, CCL22/MDC, S100A9, IL-4Ra, CD25/IL-2ra, MMP-1, IP-10 and SAA were significantly reduced while CCL2/MCP-1 increased in both groups after 56 days.

Next, we investigated the association between eicosanoid and cytokine levels in plasma (**Figure 4B**). Interestingly, CXCL9/MIG showed a diverse relationship with products of the two eicosanoid pathways, with a weak positive correlation with PGE2 (r = 0.373, p = 0.050) and a moderate negative correlation with LXA4 (r = -0.497, p = 0.007), 13-HODE (r = -0.417, p = 0.031), 14-HDoHE (r = -0.630 p = 0.0001), 12-HETE (r = -0.552, p = 0.003) and 5-HETE (r = -0.372, p = 0.056). Further, LXA4 correlated positively with Pentraxin 3 (r = 0.369, p = 0.053) and IL-18 (r = 0.0389, p = 0.040). The LOX-products 15-HETE, 12-HETE and 5-HETE all showed positive correlations with CCL1, Pentraxin3, CD25/IL-2ra and IP-10, respectively.

### Signaling Pathways in Peripheral Monocytes Induced by Lipopolysaccharide and Mycobacterial Antigens

**Figure 5:**
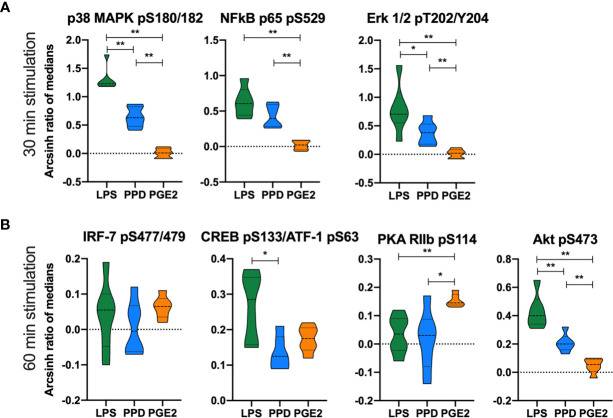
Differential phosphorylation responses in monocytes to *in vitro* stimulation with LPS, PPD and PGE2. Phosphorylation intensities measured as arcsinh ratio of medians from patients receiving standard TB treatment for 14 days (n=6) after stimulation with LPS (green), PPD (blue) and PGE2 (orange) for **(A)** 30 min and **(B)** 60 min. Significance calculated with Mann Whitney test *p < 0.05, **p < 0.01. Violin plot displaying line at median and quartiles.

Lipopolysaccharide and mycobacterial antigens bind TLRs in monocytes and induce signaling cascades with immune-modulatory effects. Thus, to further explore targets for HDT in a pilot study, we analyzed by phospho-flow cytometry the phosphorylation patterns in peripheral blood monocytes from another prospective cohort of TB patients before start of TB therapy (**Supplementary Figure S6**). We detected several phospho-epitopes following *in vitro* stimulation with endotoxin lipopolysaccharide (LPS) and mycobacterial antigens (purified protein derivative, PPD), but no effects on phosphorylation was observed by adding COX-2i to the cell cultures (**Supplementary Figure S6**). We then investigated the same phospho-epitopes during stimulation with either PGE2, LPS or PPD in samples collected from the TBCOX2 study after 14 days of standard TB treatment (**Figure 5**). *In vitro* stimulation by LPS induced phosphorylation of p38 MAPK (pS180/S182), Erk1/2 (pT202/Y204), Akt (pS473) with significantly higher intensities compared to PPD and PGE2 stimulation. In contrast, PGE2 induced higher intensity of PKA RIIb (pS114) phosphorylation than LPS and PPD as expected (**Figure 5**), indicating that LPS and PPD induce similar signaling cascades while PGE2 induce distinct pathways involving PKA. A schematic overview of the signaling pathways and the potential targets is illustrated in **Supplementary Figure S7**.

### Adjunctive COX-2i Influences Phosphorylation in Peripheral Monocytes

**Figure 6:**
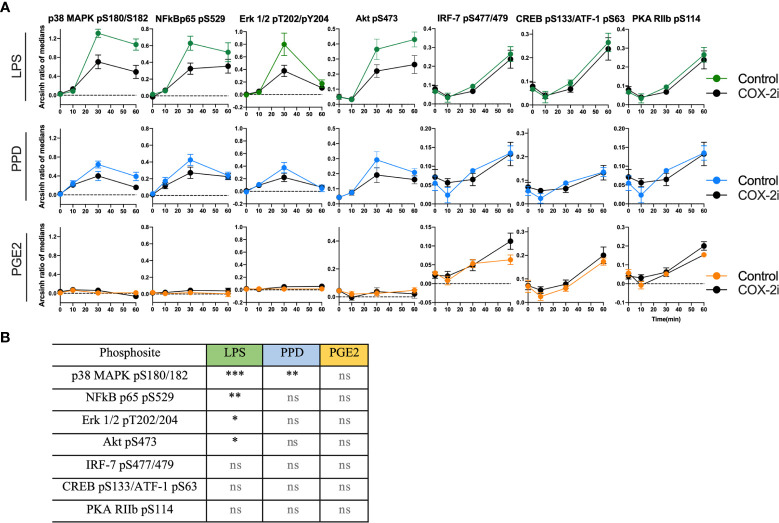
Distinct signal intensities in TB patients receiving COX-2i as adjunctive treatment. **(A)** Phosphorylation intensity induced by LPS (green), PPD (blue) and PGE2 (orange) in comparing COX-2i group (n = 8, black circles) and controls (n = 6, colored circles) after 0, 10, 30 and 60 min of stimulation. Phosphorylation intensity is measured as archsin ratio of medians. **(B)** Table of statistically significant differences between phosphorylation of the various sites in the control and COX-2i group after LPS, PPD and PGE2 stimulation. Significance calculated with multiple comparison with Holm Sidak’s correction, asterix indicate significance p-value (*p < 0.05, **p < 0.01, ***p < 0.001, ns: not significant). Error bars indicate Mean±SEM.

The phosphorylation signaling pathways are upstream of the transcription of pro-inflammatory cytokines. Therefore, we investigated if adjunctive COX-2i for 14 days influenced monocyte signaling and responsiveness to *Mtb* antigens in TB patients from the TBCOX2 trial. The phosphorylation kinetics of p38 MAPK (pS180/S182), NF*κ*B (pS529), Erk1/2 (pT202/Y204), Akt (pS473), CREB (pS133)/ATF-1 (pS63) and IRF-7 (pS477/479) showed a similar pattern in these patients as that seen in the pilot study (**Supplementary Figure S6**). Overall, we observed lower levels of phosphorylation in the COX-2i group compared to controls, especially pronounced for LPS induced phosphorylation of p38 MAPK (pS180/S182) (p<0.001), NF*κ*B (pS529) (p<0.01), Erk1/2 (pT202/Y204) (p<0.05) and Akt (pS473) (p<0.05) (**Figure 6A**). The intensity of PPD-induced phosphorylation of p38 MAPK (**Figures 6A, B**) was significantly lower in the COX-2i group compared to controls. Interestingly, the phospho-sites that were induced by PGE2 (IRF-7 (pS477/479), CREB (pS133)/ATF-1 (pS63) and PKA RIIb (pS114) displayed more pronounced phosphorylation in the COX-2i-group compared to controls, although differences were not significant.

## Discussion

Targeted immunomodulating therapy may improve TB treatment strategies. COX-2i could possibly reduce excess inflammation and tissue damage in chronic stages of TB infection with clinical benefits for patients. Still, for some patients this might come at a cost of reduced bacterial clearance due to reduced pro-inflammatory responses (Kroesen et al., 2017). In the context of a phase I/II clinical trial assessing the safety of COX-2i given adjunctive to standard TB treatment we observed that the LOX-derived products LXA4 and 12-HETE were associated with disease severity at diagnosis. Several eicosanoid metabolites were significantly reduced after 56 days of standard TB treatment, some already after 14 days, with a possible accelerated effect of COX-2i. Independent of COX-2i, pro-inflammatory plasma cytokines were reduced during the first two months of standard TB treatment, many already after 14 days while experiencing clinical improvement. Of interest, CXCL9/MIG and procalcitonin were significantly reduced only in the COX-2i-group indicating a possible adjunctive effect of COX-2i. In our *in vitro* monocyte signaling assay, LPS and mycobacterial antigens induced phosphorylation of the same phospho-epitopes. However, our findings suggest that IRF-7 is not activated by mycobacterial antigens, but rather by eicosanoids such as PGE2. We show novel data that TB patients treated with adjunctive COX-2i displayed an overall lowered signaling potential by LPS and PPD induced phosphorylation compared to controls suggesting reduced transcription of inflammatory cytokines in monocytes.

Clinical markers of TB disease severity and bacterial burden such as cavitary disease and number of days to *Mtb* positive culture (TTP) were both associated with levels of the LOX-derived metabolites LXA4, 12-HETE and 8-HETE in plasma. A detrimental role of 5/12-LOX derived metabolites in TB have been suggested due to LXA4-mediated necrosis of macrophages (Chen et al., 2008; Behar et al., 2010). Further, 12/15-LOX-derived products found in cavitary lesions may facilitate mycobacterial spread by driving neutrophilic inflammation, granuloma disintegration and tissue damage contributing to bacterial dissemination (Chen et al., 2008; Divangahi et al., 2010; Divangahi et al., 2013; Lau et al., 2015; Mishra et al., 2017). Our data are in accordance with a previous study reporting no association between PGE2 and TB disease severity, but rather an increase of LXA4, 15-epi LXA4 and LTB4 in cavitary TB disease correlating to bacterial burden (Pavan Kumar et al., 2019).

We further evaluated the effect of adjunctive COX-2i in TB patients by measuring plasma eicosanoids levels. We show that selective COX-2 inhibition influences the LOX arm of the eicosanoid system as several LOX-metabolites (LXA4, 15-HETE, 12-HETE, 8-HETE and 14-HDoHE) were reduced in the COX-2i-group, but not in controls. Our findings suggest possible beneficial effects of reduced levels of unfavorable LOX-metabolites, although it has previous been shown that COX-2 inhibition may also increase the activity of LOX-enzymes and its products (Dennis and Norris, 2015). Surprisingly, we observed no effect of COX-2i on PGE2 levels in plasma. This might be due to a suboptimal inhibitor dose or that 14 days are too early to detect possible effects. Further, we observed no association with disease severity, indicating a limited role of PGE2 than initially hypothesized in this stage of TB disease (Rangel Moreno et al., 2002). Therefore, selectively targeting the LOX-products by LOX inhibitors such as Zileuton or MK886 might be a better approach to avoid inhibiting potentially beneficial effects of the COX-2/PGE2 axis (Kaul et al., 2012; Sorgi et al., 2020).

A balanced and timely coordinated cytokine response is paramount in host immune defenses. Elucidating the systemic inflammatory milieu could expose novel HDT targets (Cicchese et al., 2018) as well as biomarkers for disease severity and treatment efficacy (Walzl et al., 2014; Kumar et al., 2019). We and others have previously reported that CCL1 and IP-10 could serve such a purpose (Tonby et al., 2015; Wergeland et al., 2015; Xiong et al., 2016; Kumar et al., 2019). Intriguingly, the cytokines pentraxin 3, IL-18, CD25 (IL-2R) and IP-10 associated with TB disease severity, were positively correlated with LOX-derived metabolites. The levels of several of these pro-inflammatory cytokines were reduced after 56 days of TB treatment, independent on COX-2i intervention, indicating that standard TB treatment is the main contributor to reduced bacterial load and inflammation. Still, there was reduced CXCL9/MIG and procalcitonin after 56 days of COX-2i treatment, not found in controls. The LOX-pathway produces both pro- and anti-inflammatory mediators (Dennis and Norris, 2015) often induced simultaneously. COX-2 induction by NF*κ*B leads to conversion of 15-HETE and induction of 5-LOX, ultimately promoting lipoxin production. Thus, prolonged and excess inflammation facilitate *Mtb* survival and result in increased TB pathology (Stek et al., 2018; Vinhaes et al., 2019).

We further explored the relationship between monocytes and COX-2i by investigating monocytes signaling induced by the endotoxin LPS, that engages TLR4, and by mycobacterial antigens (PPD) that engage both TLR4 and TLR2 (Jo et al., 2007). Several of the investigated proteins, such as p38 MAPK (Balboa et al., 2013), NF*κ*B (Bai et al., 2013), and Akt (Singh and Subbian, 2018) have been suggested as therapeutic targets in TB as they confer regulatory roles of infection and inflammation (Blumenthal et al., 2002; Yadav et al., 2004; Basu et al., 2012). TNFα-induced NF*κ*B phosphorylation is crucial in conferring mycobacterial control and granuloma formation (Fallahi-Sichani et al., 2012). We observed increased NF*κ*B p65 (pS529) phosphorylation after 30 min stimulation with LPS and PPD. PGE2 stimulation was also investigated to study potential indirect effects of COX-2i, such as altered PGE2 responsiveness. PGE2 induced phosphorylation of PKA RIIb (pS114), IRF-7 (pS477/479) and CREB (pS133)/ATF-1 (pS63) but not NF*κ*B p65 (pS529), p38 MAPK (pS180/182) and Erk1/2 (pT202/Y204), indicating that PGE2 induces distinct pathways compared to LPS and/or PPD stimulation. Bound to transmembrane EP receptors, PGE2 induce accumulation of cAMP and thus activation of the PKA signaling pathway (Diaz-Munoz et al., 2012) while IRF-7 has a multifaceted role in *Mtb* infection as it can either promote or impair pathogen control (Manca et al., 2005; Mayer-Barber et al., 2011). Our findings suggest that PGE2 rather than LPS and PPD activate IRF-7 and PKA.

To the best of our knowledge, we present for the first time novel data on the effects of COX-2i on phosphorylation patterns in peripheral blood monocytes from TB patients harvested 14 days following initiation of adjunctive COX-2i. In line with already known anti-inflammatory properties of COX-2i (Williams et al., 1999), we observed reduced LPS-induced phosphorylation of p38 MAPK (pS180/182), NF*κ*B p65 (pS529), Erk1/2 (pT202/Y204) and Akt (pS473) in the COX-2i-group possibly indicating reduced responsiveness of monocytes in patients treated with COX-2i. As several of these signaling pathways regulate transcription of pro-inflammatory cytokines (Jo et al., 2007), adjunctive COX-2i potentially reduces pro-inflammatory responses in monocytes. However, whether this reduction is beneficial or detrimental for the patients with chronic TB must be further explored. A trend of higher PGE2-induced phosphorylation was observed in the COX-2i-group compared to controls, indicating COX-2i-driven susceptibly for PGE2 in monocytes. A possible explanation is a rescue mechanism to maintain PGE2 effects in the cells possibly by upregulation of EP receptors on the cell surface (Nishimura et al., 2013). This could also explain why we observed no effect of adjunctive COX-2i on plasma PGE2 levels. The mechanism could be upregulation of EP receptors with a simultaneous lowered ability to phosphorylate components of LPS and/or PPD induced pathways.

The major limitation of our study is the small sample size due to the phase I clinical trial design. Thus, our study is exploratory and the results hypothesis generating concerning possible effects of COX-2i on the eicosanoid pathways and monocytes in TB. Also, different tissue compartments must be studied to increase the understanding of eicosanoid metabolites and cellular interplay in TB pathogenesis. Future investigations on the effects of LOX-inhibitors on cell signaling and eicosanoid pathways are needed, to illuminate their potential role as HDT-targets. In addition, the potential efficacy of both COX-2 and LOX inhibitors as adjunctive HDT in TB should be investigated in larger patient cohorts with various clinical presentations where modest differences in cell behavior can be detected.

In conclusion, we show that LOX-derived products are associated with disease severity in untreated TB, while PGE2 seem to play a less important role during the first 14 days of TB treatment. While COX-2i primarily targets the prostaglandin pathways we observed an early reduction in potentially harmful effects of LOX-derived products. COX-2i seemed to reduce pro-inflammatory responses reflected in reduced phosphorylation potential and signal transduction in monocytes. These data provide knowledge on the possible benefits and disadvantages of using adjunctive COX-2i as an HDT strategy in TB disease.

## Data Availability Statement

The datasets presented in this article are not readily available because of the privacy of the research participants included in the study. Requests to access the datasets should be directed to Professor AM-DR, email: a.m.d.riise@medisin.uio.no.

## Ethics Statement

The studies involving human participants were reviewed and approved by The Regional Committees for Medical and Health Research Ethics (REK SØ 2015/692, EudraCT nr: 2014-004986-26). The patients/participants provided their written informed consent to participate in this study.

## Author Contributions

Study concept and design, AM-DR, KTa, and MJ. Funding, AM-DR. Recruitment of participants, KTo, SJ, and AM-DR. Laboratory analyses and acquisition of data, multiplex (HCDA), flow cytometry (MJ and KN), LC-MS (EL and JN). Statistical analyses MJ and KN. Interpretation of data, MJ, KN, KTo, SJ, RM, KTa, DK, and AM-DR. Drafting of the manuscript, MJ and KN. Critical revision of the manuscript and intellectual content: KTo, SJ, AM-DR, RM, KTa, DK, HA, EL, and JN. All authors contributed to the article and approved the submitted version.

## Funding

This work was funded and supported by The Research Council of Norway (GlobVac no 234493), South Eastern Norway Regional Health Authority, Oslo University Hospital and University of Oslo.

## Conflict of Interest

The authors declare that the research was conducted in the absence of any commercial or financial relationships that could be construed as a potential conflict of interest.
