# Supplementary material for: Plasma LOX-Products and Monocyte Signaling Is Reduced by Adjunctive Cyclooxygenase-2 Inhibitor in a Phase I Clinical Trial of Tuberculosis Patients
Source: Front Cell Infect Microbiol. 2021 Jul 9;11:669623. doi: 10.3389/fcimb.2021.669623 (PMC8299478; doi:10.3389/fcimb.2021.669623)
Supplement: Supplementary file 1 [file DataSheet_1.docx]

***Supplementary Material***

**Supplementary Figure legends**

**Figure S1: Flow chart of patients included in different assays.** Overview of patent samples from the TBCOX2 cohort included in the various analysis.

**Figure S2: Phosphoflow gating strategy.** Illustration of the gating strategy used in phosphoflow experiments. Briefly, PBMC and the monocyte population is gated using forward and side scatter dot plot to remove debris and unwanted cells. Then each combination of 4 different concentration of Pacific Blue and Pacific Orange is determined. The data is then split according to population (using the Cytobank software) and the population is further gated on monocytes (HLA-DR+, CD14+) in a two-dimensional dot plot and signal from the seven different phospho-epitopes are quantified, measured as Archsin ratio of medians.

**Figure S3: Baseline eicosanoid profile in patients with low or high symptom score.** Levels of A) PGE2, LXA4 and PGE2/LXA4 ratio and B) eicosanoid derivatives comparing patients with low (circle) or high (triangle) symptom score in TB patients at diagnosis. Significance calculated with Mann Whitney T test. ns: not significant.

**Figure S4: Change in levels of eicosanoids in patients after 14 and 56 days of treatment.** A) The difference (day 14 - baseline) in levels of PGE2 and LXA4 in patients receiving either standard therapy alone (n = 16, black circle) or in combination with adjunctive treatment (n =18, open circle) for 14 days. B) Changes (day 14 - baseline) in levels of eicosanoid derivatives in patients receiving either standard therapy alone (n = 10, black circle) or in combination with adjunctive treatment (n =18, open circle) for 14 and 56 days. Significance calculated with Mann Whitney U test for unpaired data. ns: not significant.

**Figure S5: Correlation of cytokines and clinical parameters.** Cytokine levels measured in plasma was correlated with ESR (n=37), ML ratio (n=37), Ct values (n=11) and TTP (n=30). Rho were calculated using Spearman correlation. * = p <0.05, ** = p<0.01, *** = p<0.001, **** = p<0.0001.

**Figure S6: Phosphorylation pattern in monocytes from active TB patients.**

In the pilot study, signaling in monocytes in peripheral blood from active pulmonary TB patients before start of anti-TB therapy (n=5) was investigated. PBMC were either unstimulated or stimulated with LPS, PPD or PPD in combination with 20uM indomethacin (COX-1/2 inhibitor) for 0, 10, 30 and 60min. A) Heatmap displaying phosphorylation pattern of phospho-epitopes on p38MAPK, NFkB, IRF-7, Erk1/2, CREB and Akt in HLADR+, CD14+ monocytes. B) Graphical illustration of phosphorylation intensity measured as Arcsinh ratio of medians.

**Figure S7: Schematic overview of signaling pathways downstream of LPS, PPD and PGE2 signaling.** LPS are known to bind TLR2, which induced downstream phosphorylation of p38MAPK, Erk 1/2, NFkB and Akt. Phosphorylation of IRF-7, CREB and PKA RIIb was also induced by LPS although with lower intensity. PPD is known to bind several TLRs and induces phosphorylation of p38MAPK, Erk 1/2, NFkB and Akt in a similar pattern as LPS although with lower intensity. Phosphorylation induced by PGE2 showed a distinct pattern compared to both LPS and PPD. PGE2 induced phosphorylation only in phospho-sites of IRF-7, CREB and PKA RIIb.

**Supplemental table 1: Antibodies used in flow cytometry**

| **Antibody** | **Fluorochrome** | **Manufactuer** | **Cat nr** | **Volume per 100uL** |
| --- | --- | --- | --- | --- |
| HLA-DR | FITC | BD | 307604 | 2.5 |
| CD14 | PE | BD | 345785 | 2.5 |
| P38 MAPK (pS180/S182) | Alexa 647 | BD | 612595 | 2.5 |
| NFkB p65 (pS529) | Alexa 647 | BD | 558422 | 2.5 |
| IRF-7 (pS477/479) | Alexa 647 | BD | 558630 | 2.5 |
| Erk ½ (pT202/pY204) | Alexa 647 | BD | 612593 | 2.5 |
| CREB (pS133) | Alexa 647 | BD | 558434 | 5 |
| Akt (pS473) | Alexa 647 | BD | 560343 | 2.5 |
| PKA RIIb | Alexa 647 | BD | 560205 | 2.5 |
| **Barcoding reagents** |  |  |  |  |
| Pacific Blue | Pacific Blue | Thermo fisher | P10163 | 5 (100, 25, 6.25, 0.69 ng/mL) |
| Pacific Orange | Pacific Orange | Thermo fisher | P30253 | 5 (625, 156, 39, 4.3 ng/mL) |

**Figure S1**

**Figure S2**

**Figure S3**

**Figure S4**

**Figure S5**

**Figure S6**


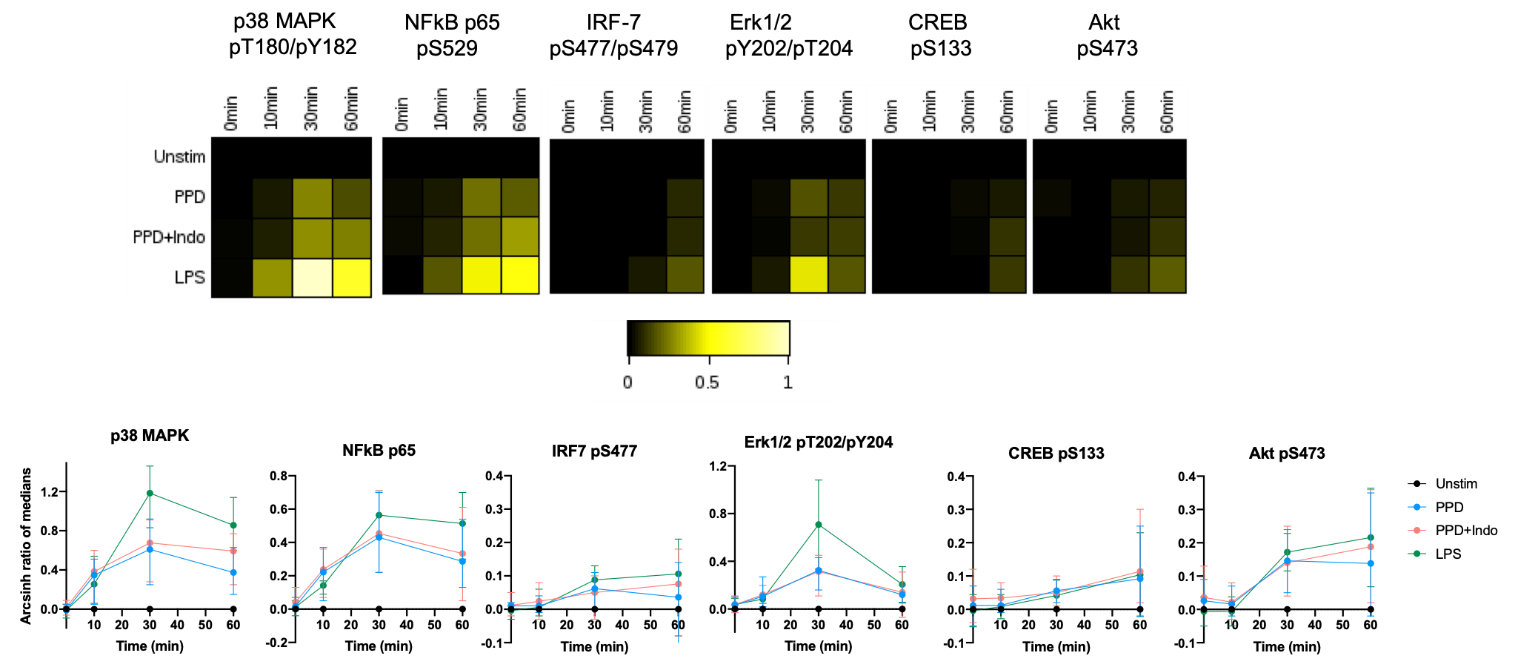


**Figure S7**
